# Exosomes promote hFOB1.19 proliferation and differentiation via LINC00520

**DOI:** 10.1186/s13018-023-04021-y

**Published:** 2023-07-29

**Authors:** Jin Wu, Licheng Zhang, Hui Liu, Jinhui Zhang, Peifu Tang

**Affiliations:** 1 Medical School of Chinese PLA, Chinese PLA General Hospital, No. 28 Fuxing Road, Haidian District, Beijing, 100853 China; 2 Department of Orthopedics, National Clinical Research Center for Orthopedics, Sports Medicine and Rehabilitation, Chinese PLA General Hospital, Beijing, 100853 China; 3 Department of Orthopedics, The 909th Hospital, School of Medicine, Xiamen University, Zhangzhou, 363000 China

**Keywords:** Osteoblast, Osteoporosis, Human umbilical cord mesenchymal stem cell-derived exosomes, LINC00520

## Abstract

**Background:**

Osteoporosis remains a significant clinical challenge worldwide. Recent studies have shown that exosomes stimulate bone regeneration. Thus, it is worthwhile to explore whether exosomes could be a useful therapeutic strategy for osteoporosis. The purpose of this study was to investigate the effects of exosomes derived from human umbilical cord mesenchymal stem cells (hucMSCs) on osteoblast proliferation and differentiation.

**Methods:**

Exosomes were isolated from hucMSCs. Bioinformatics analysis was performed to identify the differentially expressed lncRNAs in myeloma-derived mesenchymal stem cells. Plasmids encoding LINC00520 or short hairpin RNA of LINC00520 were transfected into hucMSCs and then exosomes were isolated. After human osteoblasts hFOB1.19 were exposed to the obtained exosomes, cell survival, cell cycle, apoptosis and calcium deposits of hFOB1.19 cell were detected by MTT, 7-aminoactinomycin D, Annexin V-FITC/propidium iodide and Alizarin red staining, respectively.

**Results:**

In hFOB1.19 cells, 10 × 10^9^/mL hucMSC-derived exosomes inhibited cell proliferation, arrested cell cycle, and promoted apoptosis, while hucMSCs or 1 × 10^9^/mL exosomes promoted cell proliferation, accelerated cell cycle, and promoted calcium deposits and the expression of OCN, RUNX2, collagen I and ALP. In hFOB1.19 cells, exosomes from hucMSCs with LINC00520 knockdown reduced the survival and calcium deposits, arrested the cell cycle, and enhanced the apoptosis, while exosomes from hucMSCs overexpressing LINC00520 enhance the proliferation and calcium deposits and accelerated the cell cycle.

**Conclusions:**

LINC00520 functions as a modulator of calcium deposits, and exosomes derived from hucMSCs overexpressing LINC00520 might be a novel therapeutic approach for osteoporosis.

**Supplementary Information:**

The online version contains supplementary material available at 10.1186/s13018-023-04021-y.

## Background

Osteoporosis is a major medical and socioeconomic challenge worldwide, which is characterized by systemic damage to bone mass and microstructure that increases the risk of fragility fractures [1]. Osteoblasts form mineralized bone by producing calcium- and phosphate-based minerals [2]. Thus, direct upregulation of osteoblast proliferation and activity could be an effective way to stimulate bone regeneration and mitigate osteoporosis. Moreover, exosomes have been shown to play important roles in the regulation of osteoblast proliferation and activity. Therefore, mesenchymal stem cell (MSC)-derived exosomes may regulate osteoblast differentiation.

Exosomes, 40–160 nm vesicles generated via the endosomal pathway, are secreted by a variety of cells and play a key role in cell–cell communication [3]. Exosomes contain DNA, coding and noncoding RNA, proteins, and antigen presentation molecules that are shuttled between cells [3–7]. Studies have shown that MSC-derived exosomes can induce the differentiation of naïve stem cells into an osteogenic lineage [8]. Exosomes derived from prostate cancer cells can robustly increase human osteoblast proliferation [9], and exosomes derived from human osteosarcoma have been shown to promote the formation of calcium deposits and increase alkaline phosphatase (ALP) activity of osteoblast [10]. Exosomes have been shown to play important roles in the regulation of osteoblast proliferation and activity. Nowadays, emerging studies are focusing on exosomes derived from human umbilical cord mesenchymal stem cell. Human umbilical cord mesenchymal stem cells (hUCMSCs) possess the ability to differentiate into multiple cell types, a property known as pluripotency or multipotency [11]. This differentiation process involves the induction of hUCMSCs with specific growth factors, cytokines, or chemical compounds, which activate signaling pathways and modulate gene expression. Various protocols have been developed to differentiate hUCMSCs into specific lineages such as osteoblasts, adipocytes, chondrocytes, myocytes, and neurons [12–14]. However, whether exosomes from human umbilical cord mesenchymal stem cell (hucMSC-exosomes) exert protective effects against osteoporosis is unknown.

Long non-coding RNAs (lncRNAs) have emerged as novel modulators of MSC osteogenesis [15–17]. Studies showed that MALAT1 delivered by bone marrow-derived MSC-secreted exosomes could alleviates osteoporosis [18], and knockdown of lncRNA BCAR4 was reported to alleviate osteoporosis [19]. The lncRNAs SNHG1, LINC00963, and HOTAIRM1 promoted osteogenic differentiation [20–22]. LINC00520 is a lncRNA that is widely expressed in various tissues and has been linked to the clinicopathological characteristics of 11 cancers [23]; however, its role in osteoblast differentiation has not been explored.

In our study, we co-cultured hucMSC-exosomes with hFOB1.19 human osteoblast cells to explore how these exosomes influence the proliferation and calcium deposits of osteoblasts. Using bioinformatic analysis, we identified LINC00520 as the most highly expressed lncRNA in myeloma-derived MSCs. Silencing and overexpression of LINC00520 showed that LINC00520 plays a crucial role in promoting osteoblast calcium deposits.

## Methods

### Cell lines

The human osteoblast cell line hFOB1.19 and human umbilical cord mesenchymal stem cells (hucMSCs) were obtained from Xiamen Immocell Biotechnology Co., Ltd., and were maintained in dulbecco's modified eagle medium (DMEM)/F12 (catalog number: 12500062, Gibco, Grand Island, USA) supplemented with 10% fetal bovine serum (FBS, catalog number: 10099141C, Gibco) and 1% penicillin–streptomycin (catalog number: 15140148, Gibco). All cells were incubated at 37 °C and 5% CO_2_.

### Plasmids

The plasmids encoding short hairpin ribonucleic acid (shRNA) of LINC00520 (shLINC00520), the negative control of shLINC00520 (shNC), LINC00520 (LINC00520 OE), or the negative control of LINC00520 OE (Vector) were obtained from Xiamen Antihela Biotechnology Co., Ltd (Xiamen, China). For the construction of plasmids, the shRNA targeting LINC00520 has been inserted into the pLKO.1-TRC vector at the AgeI and EcoRI restriction sites. Furthermore, LINC00520 has been inserted into the plv-cmv-mcs-pGK-puro vector using the EcoRI and BamHI restriction sites. The primers used for plasmid construction are listed in Table 1.

**Table 1:** Primers for plasmid construction and RT-qPCR

Primers		Sequence (5′-3′)
shLINC00520	F	CCGGGGACTGCAATTCACAAGAACACTCGAGTGTTCTTGTGAATTGCAGTCCTTTTT
	R	AATTAAAAAGGACTGCAATTCACAAGAACACTCGAGTGTTCTTGTGAATTGCAGTCC
LINC00520 OE	F	ATTTCGATTTCTTGGCTTTA
	R	TTCTTTCCCCTGCACTGTAC
HEIH	F	GCAGTAACAGAGTCAACA
	R	CAAGAATTATCGTGGAACAG
LINC00520	F	GTAAGATATGACTGTGCTC
	R	CATACTCATGGAGTTCCA
LINC00623	F	CAGTTGTTCCAGCATAGTG
	R	ATGTATCCCAGAAACCCTAAA
OIP5-AS1	F	AATCAGCAGAGGACCATT
	R	CAGCATCACAGCATTCAT
PRR34-AS1	F	GCAGGAATATGACAACAG
	R	GTTATTAAGATGGTAGCAGTT
18S	F	CGACGACCCATTCGAACGTCT
	R	CTCTCCGGAATCGAA CCCTGA
OCN	F	CGCTACCTGTATCAATGGCTGG
	R	CTCCTGAAAGCCGATGTGGTCA
RUNX2	F	CCCAGTATGAGAGTAGGTGTCC
	R	GGGTAAGACTGGTCATAGGACC
Collagen I	F	GATTCCCTGGACCTAAAGGTGC
	R	AGCCTCTCCATCTTTGCCAGCA
ALP	F	AGCGTGACTTGAAGTGTTGCAT
	R	GAAAGGACCTGGACCACACAGA

F, forward primer; R, reverse primer

### Identification of hucMSCs

To evaluate the surface markers of hucMSCs, hucMSCs at passage three were incubated with antibodies anti-CD34 (catalog number: 343503, BioLegend, San Diego, CA, USA), anti-CD73 (catalog number: 344015, BioLegend), anti-CD29 (catalog number: 303004, BioLegend), anti-CD14 (catalog number: 397706, BioLegend), anti-CD105 (catalog number: 323203, BioLegend), anti-CD19 (catalog number: 302205, BioLegend), anti-CD45 (catalog number: 304005, BioLegend), anti-HLA-DR (catalog number: 327005, BioLegend), and anti-CD90 (catalog number: 328108, BioLegend). Fluorescence was detected using a flow cytometer (NovoCyte 1300; ACEA, San Diego, CA, USA) to identify hucMSCs.

To evaluate cell differentiation, hucMSCs at passage three were cultured in adipogenic (catalog number: A1007001, Gibco, Grand Island, USA), chondrogenic (catalog number: A1007101, Gibco), or osteogenic medium (catalog number: A1007201, Gibco) for 3 weeks. Subsequently, the cells were fixed with 4% paraformaldehyde, and stained with oil red O (catalog number: HY-D1168, MedChemExpress, New Jersey, USA), alcian blue (catalog number: HY-D0001, MedChemExpres), or alizarin red (catalog number: HY-120601, MedChemExpres), respectively. The stained cells were observed under a light microscope (Olympus, Tokyo, Japan).

### Extraction, purification, and characterization of hucMSC-exosomes

Exosomes were extracted from the hucMSCs as follows. The hucMSCs were cultured in medium containing 10% FBS which was centrifuged at 100,000 × *g* for 18 h to remove exosomes. After 24 h, the medium was changed to DMEM/F12 without FBS. After one hour, 5 µg plasmid (shLINC00520, shNC, LINC00520 OE, or Vector) was transfected into hucMSCs using ExFect® Transfection Reagent (catalog number: T101, Vazyme, Nanjing, China). Six hours after transfection, the medium was replaced with DMEM/F12 containing 10% FBS. The medium was collected 48 h post-transfection to get the supernatant after centrifugation at 600 × *g* for 5 min. By centrifuging at 100,000 × *g* for 60 min using an ultra-high-speed centrifuge, the pellets were washed and resuspended with phosphate buffered solution (PBS) to obtain the hucMSC-exosomes and stored at − 80 °C until use.

### Observation of hucMSC-exosomes under the electron microscope

The prepared hucMSC-exosomes were fixed with 4% glutaraldehyde at 4 °C for 2 h, rinsed three times with 0.1 mol/L PBS and fixed in 1% osmium tetraoxide for 2 h. After gradient dehydration of conventional ethanol and acetone, the samples were immersed, embedded, and polymerized in epoxy resin, followed by preparation of 0.5 nm ultrathin sections. After optical location, 60 nm ultrathin sections were prepared, which were then stained with uranium acetate and lead citrate and observed under a JEM-1230 electron microscope (Nihon Denshi, Tokyo, Japan).

### Detection of exosome size

A total of 20 μg exosomes were dissolved in 1 mL PBS and swirled for 1 min, so that the exosomes were evenly distributed. Subsequently, the diameter and concentration of exosomes were measured by NanoSight particle tracking analyzer (NTA, Malvern Panalytical, Malvern, UK).

### Labeling hucMSC-exosomes with carboxyfluorescein diacetate succinimidyl-ester (CFSE)

CFSE (catalog number: C1031, Beyotime, Shanghai, China) was dissolved in Dimethyl sulfoxide to a concentration of 1 mM. CFSE was added to hucMSC-exosomes at a final concentration of 1 μM and incubated for 4 h at 37 °C. The medium was removed after centrifugation at 100,000 g for 90 min. Then, 10 mL of PBS was added to the exosome pellet and gently resuspended to ensure proper mixing. Following that, another centrifugation at 100,000 g for 90 min was performed to pellet the exosomes once again. Then, CFSE-labeled hucMSC-exosomes were added to hFOB1.19 cells and incubated for 48 h. The cells were stained with a 4′,6-diamidino-2-phenylindole solution (DAPI; catalog number: C1005, Beyotime). The signals were observed using a fluorescence microscope (Olympus, Tokyo, Japan).

### Co-culture of osteoblasts and hucMSCs or hucMSC-exosomes

Human osteoblasts (hFOB1.19) were seeded into the bottom chamber of 6-well Transwell plates containing DMEM/F12 supplemented with 10% exosome-free FBS at 2 × 10^4^ cells per well. hucMSCs (2 × 10^4^ cells per well), different concentrations of hucMSC-exosomes (1 × 10^9^/mL, 5 × 10^9^/mL or 10 × 10^9^/mL), or hucMSCs (2 × 10^4^ cells per well) and 10 μM GW4869, an inhibitor of exosome synthesis/release [24, 25], were added into the upper chamber. After 48 h, hFOB1.19 cells were collected for follow-up experiments.

### Immunoblotting

hFOB1.19 cells were collected and lysed in radioimmunoprecipitation assay (catalog number: P0013B, Beyotime) buffer containing protease and phosphatase inhibitor by incubation on ice for 30 min, homogenization, and sonication. The proteins were extracted, and the concentration was determined using a BCA protein assay kit (catalog number: P0011, Beyotime). Samples were then heated at 95 °C for 10 min. Equal protein (20 μg) was loaded into each well of a 12% SDS-PAGE gel and electrophoresed at 80 V for 1.5 h. The separated proteins were then transferred to PVDF membranes (catalog number: IPVH00010, Millipore, Massachusetts, USA) at 300 mA for 2.5 h. The membrane was blocked with 5% non-fat milk for 1 h and then incubated with CD81 antibody (1:4000, catalog number: 66866-1-Ig, Proteintech), CD9 antibody (1:3000, catalog number: 20597-1-AP, Proteintech), CD63 antibody (1:3000, catalog number: 25682-1-AP, Proteintech), Actin antibody (catalog number: 20536-1-AP, Proteintech, Wuhan, China), OCN antibody (catalog number: Ab133612, Abcam, Cambridge, UK), RUNX2 antibody (catalog number: 20700-1-AP, Proteintech), CoII antibody (catalog number: 27836-1-AP, Proteintech), or ALP antibody (catalog number: 13365-1-AP, Proteintech) overnight at 4 °C. The membrane was then incubated with horseradish peroxidase-linked goat anti-rabbit IgG (catalog number: SA00001-2, Proteintech) for 1 h at room temperature. After washing three times (10 min each), the ECL kit (catalog number: WP20005, Invitrogen, CA, USA) was used to detect the protein bands, which were visualized using X-ray film. The original images of all immunobloting in this study are in Additional file 1.

### MTT assay

After hFOB1.19 cells were co-cultured with hucMSCs or hucMSC-exosomes for 48 h, MTT was added to each well to a final concentration of 0.5% MTT (catalog number: 40201ES72; Yeasen Biotechnology Co. Ltd., Shanghai, China). After incubation for 3 h, the medium in each well was discarded, and 100 μL of DMSO was added. The plate was incubated on a shaker at 70–100 rpm for 15 min, and then the optical density (OD) was measured at 490 nm using a SpectraMax® Absorbance Plate Reader (supplier number: PLUS 384, Molecular Devices, San Francisco, USA).

### Cell cycle detection

The hFOB1.19 cells co-cultured with hucMSCs or hucMSC-exosomes for 48 h were detached carefully by trypsin and collected at 500 × *g* for 5 min. After fixed in ice-cold 70% ethanol (catalog number: 10009218, Sinopharm Group, Beijing, China) at − 20 °C for 12 h, the hFOB1.19 cells were incubated with 0.2% Triton X-100 (catalog number: HFH10, Invitrogen) containing 100 µg/mL RNase A (catalog number: EN0531, Thermo Scientific, MA, USA) at 37 °C for 30 min. 20 mg/mL 7-aminoactinomycin D (7-AAD, catalog number: ST515-1 mg, Beyotime) was added to each sample and incubated at 37 °C for 8 min in an incubator. Then, cells were analyzed by flow cytometry (ACEA, San Diego, CA, USA).

### Quantitative PCR (qPCR)

Total RNA was extracted from hFOB1.19 cells using TRIzol reagent (catalog number: 15596026, Invitrogen), and the extracted RNA was used for cDNA synthesis using a reverse transcription kit (catalog number: RR036A, Takara, Tokyo, Japan). qPCR was performed using ChamQ SYBR qPCR Master Mix (catalog number: Q311-02, Vazyme) and an AriaMx Real-Time qPCR system (part number: G8830A, Agilent, California, USA). The real-time PCR cycling condition were as follows: stage I: 95 °C for 30 s; stage II: 40 cycles of 95 °C for 10 s and 60 °C for 30 s; stage III: 95 °C for 15 s, 60 °C for 1 min, and 95 °C for 15 s. The primer sequences are shown in Table 1.

### Apoptosis detection

The hFOB1.19 cells co-cultured with hucMSCs or hucMSC-exosomes for 48 h were collected by centrifugation at 500 × *g* for 5 min, and stained for 20 min using an apoptosis detection kit (catalog number: A211-01, Vazyme, Nanjing, Jiangsu, China) according to the merchant instructions. Then, NovoCyte flow cytometry (ACEA, San Diego, CA, USA) and associated software (NovoExpress 1.4.1, ACEA) were used to analyze the apoptosis ratio.

### Alizarin red staining

hFOB1.19 cells were seeded into the bottom chamber of 6-well Transwell plates at 2 × 10^4^ cells per well. hucMSCs (2 × 10^4^ cells per well) or 1 × 10^9^/mL exosomes from hucMSCs transfected with plasmids (shNC, shLINC00520, Vector, or LINC00520 OE) were added into the upper chamber. After 14 days, hFOB1.19 cells were stained with 2% alizarin red (pH 4.2, Sigma-Aldrich, St Louis, MO, USA) for 10 min, and then washed with distilled water. The mineralized nodules were examined with a light microscope (Olympus, Tokyo, Japan).

### Bioinformatics analysis

LncRNA data from multiple myeloma-derived MSC was obtained from the gene expression omnibus (GSE118985, https://www.ncbi.nlm.nih.gov/geo/query/acc.cgi?acc=GSE118985) and ggplot2 (v3.3.6) was used to plot the graph. The expression levels of the top 10 lncRNAs in multiple myeloma-derived MSCs were at the intersection of lncRNAs from exosome exoRBase (http://www.exorbase.org/). Venn diagram was drawn using Venny 2.1 (https://bioinfogp.cnb.csic.es/tools/venny/index.html).

### Statistical analysis

Statistical analyses were conducted using GraphPad Prism software (version 8.0, GraphPad Software Inc., CA, USA). Student’s *t*-test (unpaired) was used to compare the differences between two groups for parametric data. One-way analysis of variance followed by Tukey's test were used for among multiple groups. Statistics were deemed significant at *P* < 0.05.

## Results

### Verification of hucMSCs and preparation of hucMSC-exosomes

**Fig. 1:**
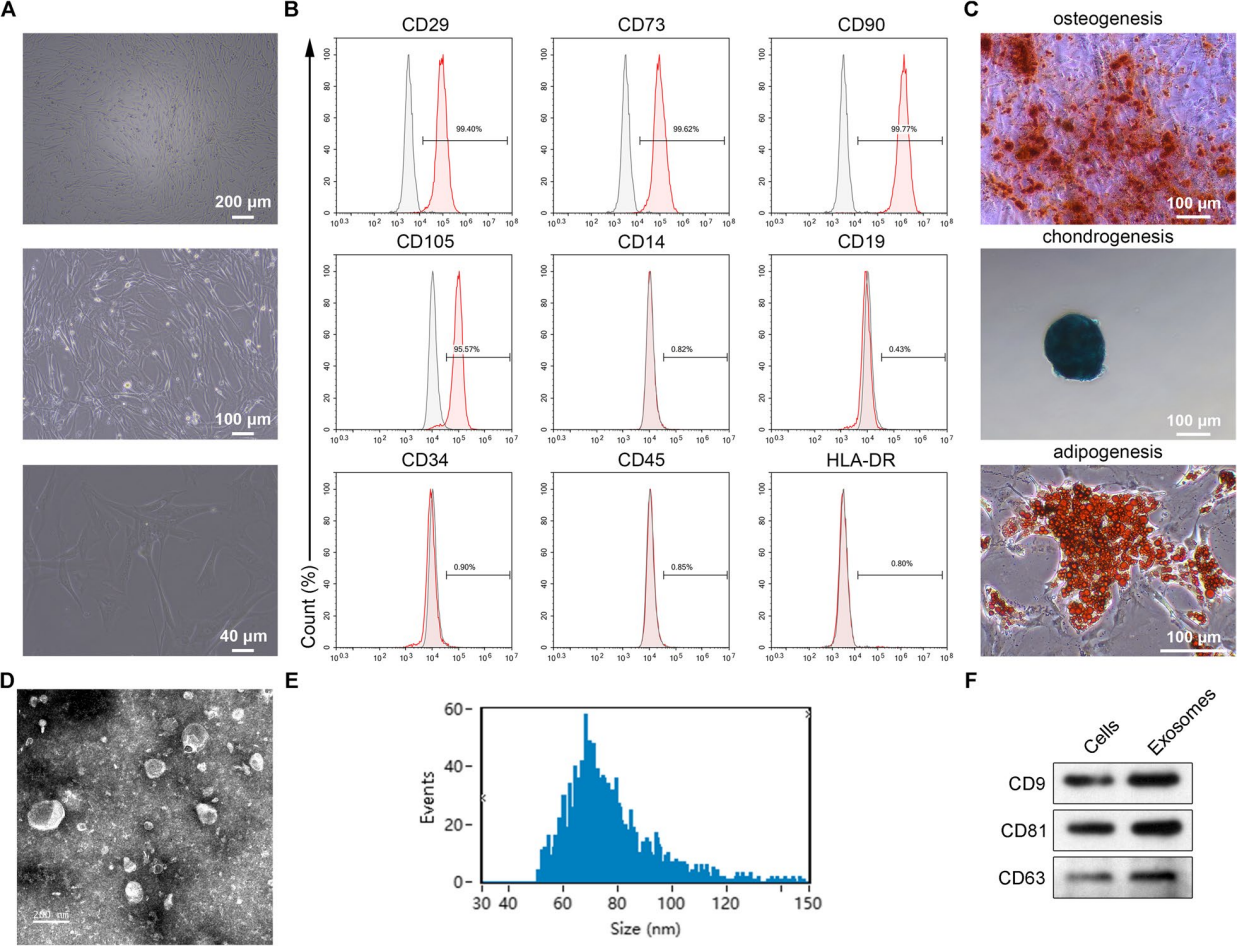
Identification of human umbilical cord mesenchymal stem cells (hucMSCs) and hucMSC-derived exosomes.** A** The morphology of human umbilical cord-derived MSCs as observed using phase-contrast microscopy. **B** Immunophenotype of human umbilical cord-derived MSCs showing expression of CD29, CD73, CD90, CD105, CD14, CD19, CD34, CD45, and HLA-DR by flow cytometry. **C** Effects of human umbilical cord-derived MSCs on osteogenesis, adipogenesis, and soft tissue formation. **D** Morphology of hucMSC-derived exosomes as determined by transmission electron microscopy. **E** Diameter distribution of hucMSC-exosomes as determined by dynamic light scattering. **F** Immunoblotting of CD9, CD81, CD63, and actin in hucMSCs and derived exosomes

We first cultured hucMSCs and observed their elongated adherent shape using phase-contrast microscopy (Fig. 1A). Next, we performed flow cytometry to confirm the immunophenotype of the hucMSCs. The surface markers CD29, CD73, CD90, and CD105 were highly expressed in hucMSCs (Fig. 1B). However, positive expression levels of CD19, CD34, CD45, and HLA-DR were not observed. The multidirectional differentiation potential of hucMSCs was confirmed by osteogenesis, adipogenesis, and chondrogenesis assays. After induction of osteoporosis, adipogenesis, and chondrogenesis, positive staining for alkaline phosphatase, Oil-Red-O, and Von Kossa was observed (Fig. 1C).

We then isolated exosomes from the supernatant of hucMSCs using a previously described method [26]. To verify their identity as exosomes, we first observed their round shape using electron microscopy (Fig. 1D). Then, we used dynamic light scattering, which showed that the size of the hucMSC-exosomes ranged from 50 to 150 nm (Fig. 1E). Finally, immunoblotting showed that CD9, CD63, and CD81 protein levels were higher in hucMSC-exosomes compared to the levels in cells (Fig. 1F). These results confirm that we cultured hucMSCs and isolated exosomes from the hucMSCs successfully.

### A low concentration of hucMSC-exosomes promotes the proliferation of osteoblasts

**Fig. 2:**
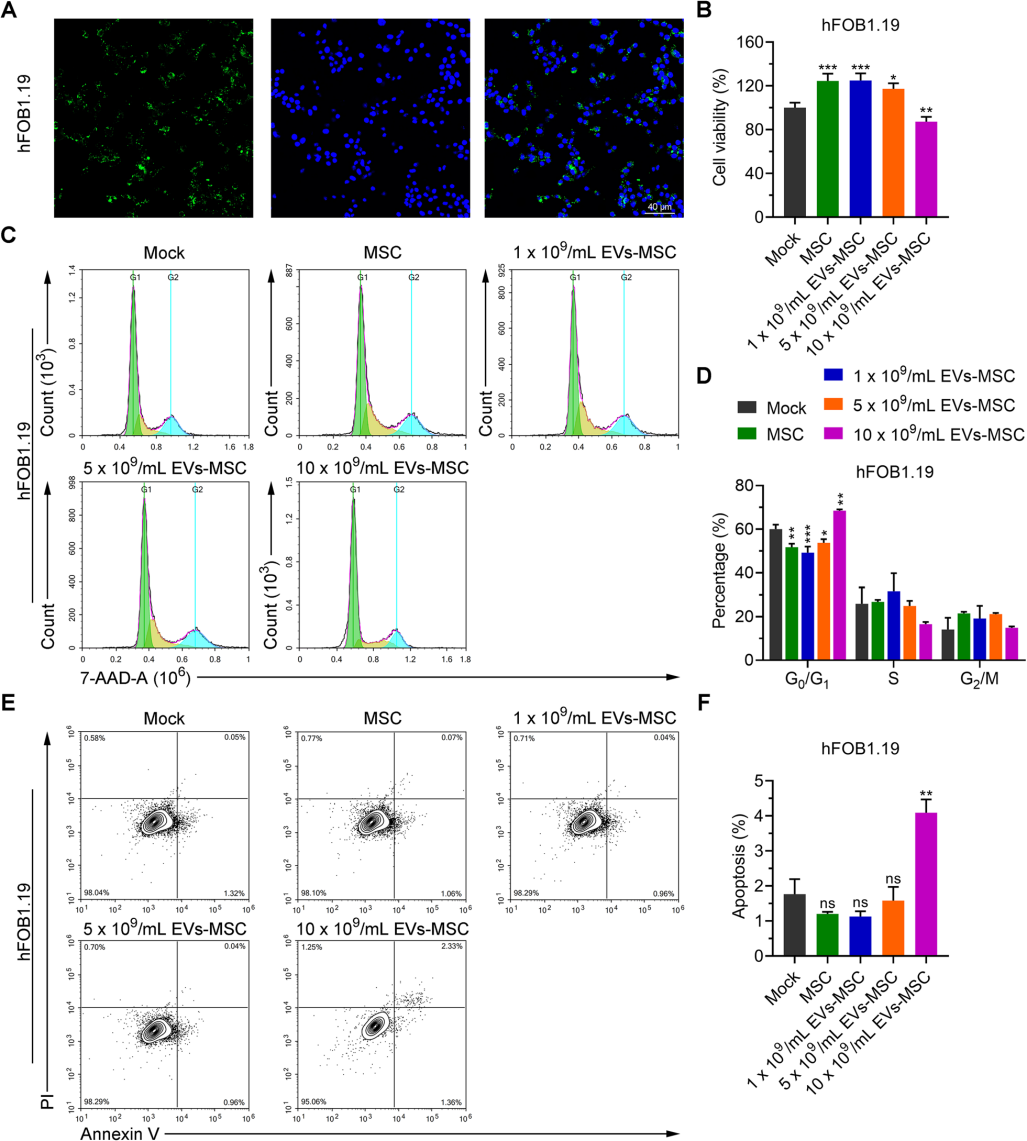
A high concentration of hucMSC-exosomes inhibited the proliferation of osteoblasts. **A** Exosomes derived from hucMSCs were labeled with CFSE and the immunofluorescence of hFOB1.19 cells treated with the exosomes is shown. **B** Viability of hFOB1.19 cells cultured with MSCs and different concentrations of hucMSC-exosomes (EVs-MSC) as determined by MTT assay. **C, D** Cell cycle analysis of hFOB1.19 cells treated with hucMSCs or different concentrations of hucMSC-exosomes. **E, F** Cell apoptosis as analyzed by FITC-Annexin V kit and flow cytometry

To explore the effect of hucMSC-exosomes on osteoblasts, we treated hFOB1.19 cells with CFSE-labeled hucMSC-exosomes and found that the exosomes could enter hFOB1.19 cells after 48 h of co-culture with CFSE-labeled hucMSC-exosomes (Fig. 2A). To determine whether hucMSCs and hucMSC-exosomes regulate the proliferation of osteoblasts, hFOB1.19 cells were co-cultured with hucMSCs or different concentrations of hucMSC-exosomes for 48 h. The results of an MTT assay indicated that cell viability was increased when cells were co-cultured with hucMSCs or the two lower concentrations of hucMSC-exosomes (1 × 10^9^/mL and 5 × 10^9^/mL) compared with control cells, but the highest concentration of exosomes (10 × 10^9^/mL) had the opposite effect and reduced viability (Fig. 2B). Consistently, when hFOB1.19 cells were co-cultured with hucMSCs or hucMSC-exosomes at lower concentrations (1 × 10^9^/mL), the number of hFOB1.19 cells in G1 phase decreased (Fig. 2C, D). After co-cultured with hucMSC-exosomes at the highest concentration (10 × 10^9^/mL), the cell cycle of hFOB1.19 cells was stagnated in G1 phase (Fig. 2C, D). Apoptosis of hFOB1.19 cells was also investigated. The apoptosis was not induced in hFOB1.19 cells cultured with hucMSCs or low-concentration hucMSC-exosomes, while higher apoptosis was induced in hFOB1.19 cells cultured with 10 × 10^9^/mL hucMSC-exosomes (Fig. 2E, F). These results suggested that high concentration of hucMSC- exosomes inhibited proliferation and induced apoptosis of hFOB1.19 cells.

### hucMSCs and hucMSC-exosomes promote calcium deposition in hFOB1.19 cells

**Fig. 3:**
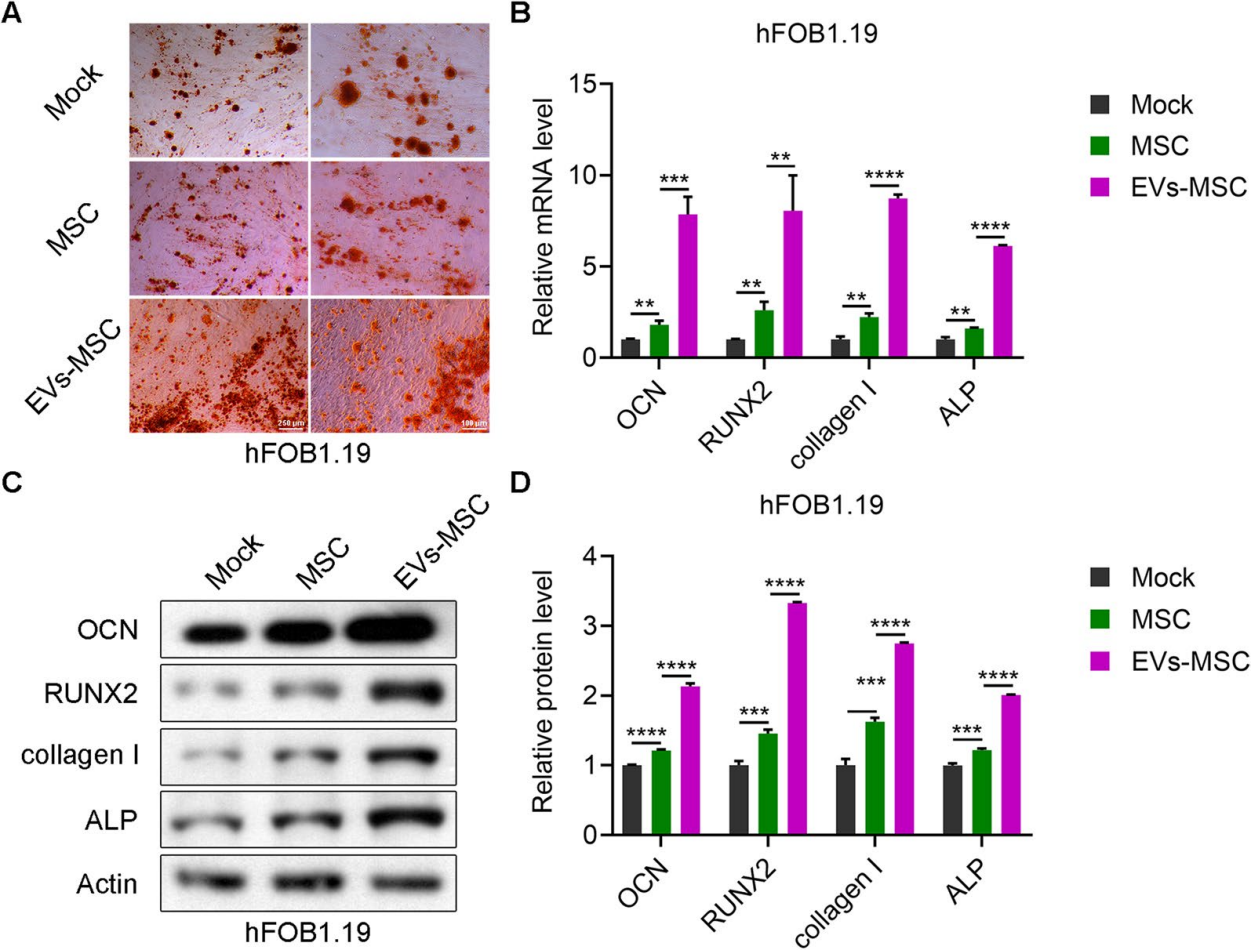
hucMSCs and hucMSC-exosomes promote osteoblast differentiation. **A** Alizarin red staining of hFOB1.19 cells treated with hucMSCs and hucMSC-exosomes. **B** The mRNA expression levels of OCN, RUNX2, Collagen I, and ALP in hFOB1.19 cells treated with hucMSCs (MSC) or hucMSC-exosomes (EVs-MSC) as detected by RT-PCR. **C, D** Immunoblotting of OCN, RUNX2, collagen I, and ALP protein levels in hFOB1.19 cells treated with hucMSCs (MSC) or hucMSC-exosomes (EVs-MSC)

Calcium content in hFOB1.19 cells after culture with hucMSCs or hucMSC-exosomes was assessed by Alizarin red staining. hFOB1.19 cells cocultured with 1 × 10^9^/mL hucMSC-exosomes showed more calcified nodules than cocultured with MSCs (Fig. 3A). The relative mRNA expression levels of marker of late differentiation of osteoblasts OCN, necessary specific transcription factor for osteogenic differentiation RUNX2, and markers of early differentiation of osteoblasts collagen I and ALP were increased in hFOB1.19 cells after co-culture with hucMSCs or hucMSC-exosomes (Fig. 3B). Consistent with the mRNA expression, the protein levels of OCN, RUNX2, collagen I and ALP were significantly upregulated in hFOB1.19 cells after co-culture with hucMSCs or hucMSC-exosomes (Fig. 3C, D). Compared with hucMSCs, hucMSCs-exosomes enhanced the expression of OCN, RUNX2, collagen I and ALP in hFOB1.19 cells (Fig. 3B–D). These findings indicate that hucMSCs and hucMSC-exosomes promote calcium deposition in hFOB1.19 cells.

### Identification and validation of the differential expressed lncRNAs in hucMSC-exosomes

**Fig. 4:**
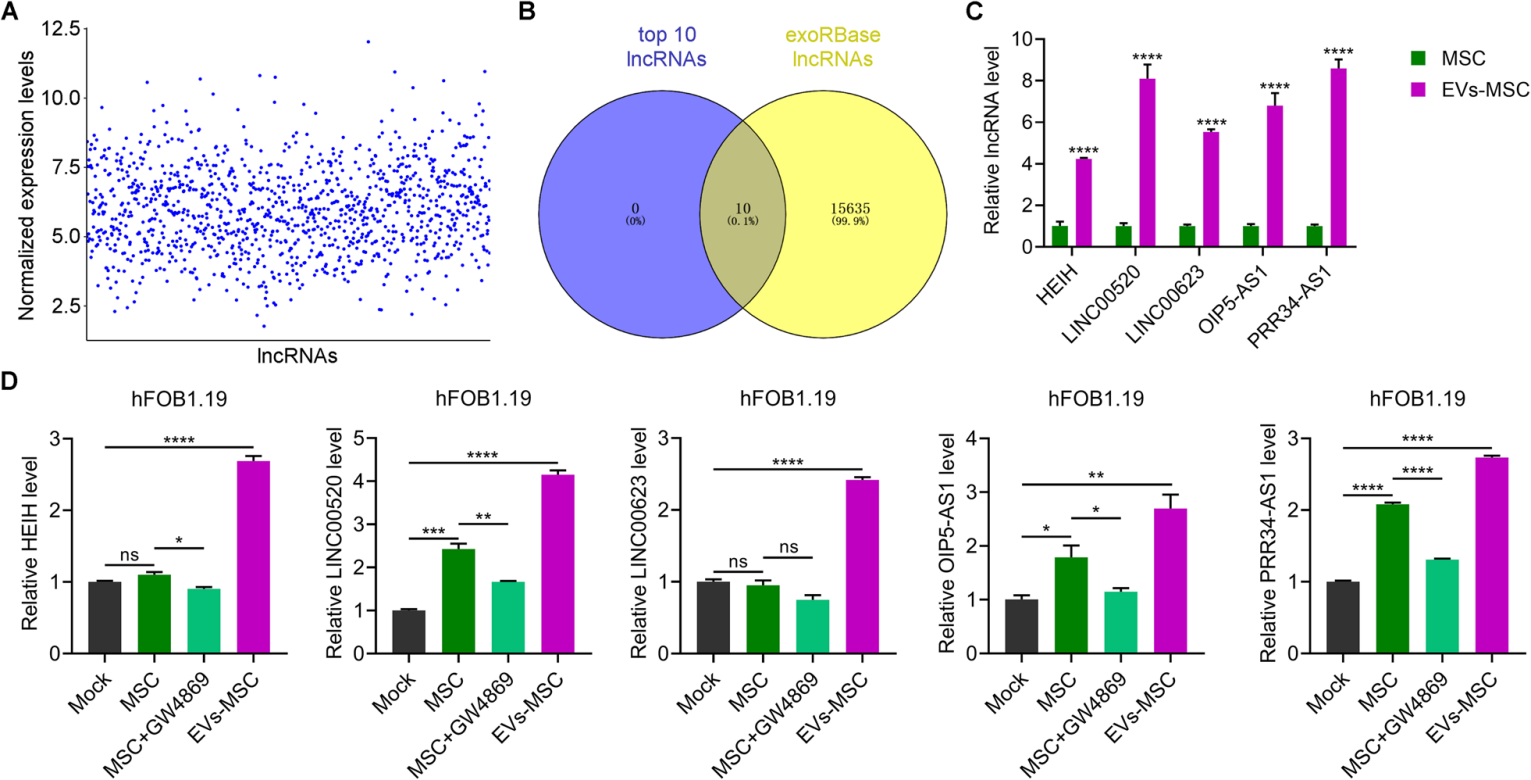
Validation of the differentially expressed lncRNAs in hucMSC-exosomes. **A** Normalized lncRNA expression levels determined using bioinformatic methods. **B** Venn diagram of the top 10 expressed lncRNAs in hucMSCs (MSC) and the lncRNAs in the exoRBase database. **C** Relative expression of the lncRNAs HEIH, LINC00520, LINC00623, OIP5-AS1, and PRR34-AS1 in hucMSCs (MSC) or hucMSC-exosomes (EVs-MSC) as measured using RT-PCR. **D** Expression of HEIH, LINC00520, LINC00623, OIP5-AS1, and PRR34-AS1 in hFOB1.19 cells treated with MSC, MSC + GW4869 and EVs-MSC

To identify the role of lncRNAs in osteoblasts, we performed a bioinformatic analysis. The normalized expression levels of the lncRNAs in the GSE118985 dataset [27] were analyzed (Fig. 4A). The top 10 highly expressed lncRNAs, HEIH, LINC00520, LINC00623, OIP5-AS1, PRR34-AS1, MALAT1, ZFAS1, SNHG19, DANCR, and ITGB2-AS1, were validated in the exoRBase database (http://www.exorbase.org/) (Fig. 4B). The levels of lncRNA HEIH, LINC00520, LINC00623, OIP5-AS1, and PRR34-AS1 were higher in hucMSC-exosomes than in hucMSCs (Fig. 4C). Further analysis showed that LINC00520, OIP5-AS1, and PRR34-AS1, but not HEIH and LINC00623, were upregulated in hFOB1.19 cells cultured with hucMSCs compared with in hFOB1.19 cells cultured without hucMSCs (Fig. 4D). GW4869 acts as an inhibitor of neutral sphingomyelinase 2 (nSMase2), an enzyme involved in ceramide synthesis [28]. Ceramide is a key lipid component of cellular membranes and plays a regulatory role in various cellular processes, including exosome biogenesis. Inhibition of nSMase2 by GW4869 leads to a reduction in ceramide levels within cells, which subsequently affects the formation and release of exosomes. GW4869 inhibited the up-regulation effect of hucMSCs on HEIH, LINC00520, OIP5-AS1, and PRR34-AS1, but not LINC00623, in hFOB1.19 cells (Fig. 4D). hucMSC-exosomes significantly increased the levels of HEIH, LINC00520, LINC00623, OIP5-AS1, and PRR34-AS1 in hFOB1.19 cells (Fig. 4D). Figure 4D illustrates that the relative expression level of LINC00520 in hFOB1.19 cells co-cultured with EVs-MSC was approximately 4-fold higher than that in hFOB1.19 cells without co-culture (Mock group), while the relative expression level of PRR34-AS1 in hFOB1.19 cells co-cultured with EVs-MSC was nearly 3-fold higher than that in hFOB1.19 cells without co-culture. Therefore, subsequent experiments were focused on the function of LINC00520 in hFOB1.19 cells.

### LINC00520 in hucMSC-exosomes enhances osteoblast proliferation

**Fig. 5:**
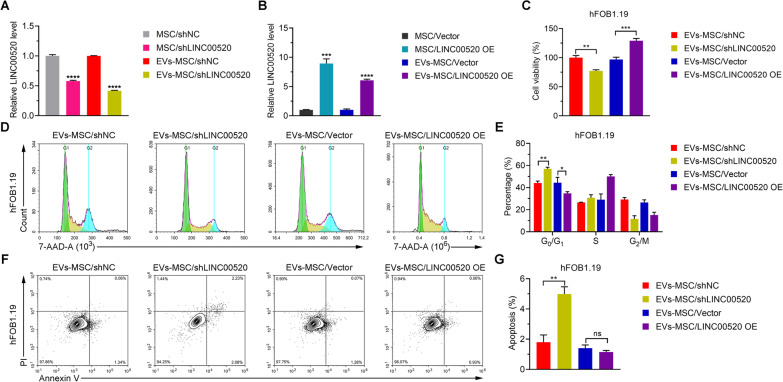
LINC00520 in hucMSC-exosomes regulates the proliferation of osteoblasts. **A** Relative LINC00520 expression levels in hFOB1.19 cells exposed to hucMSCs transfected with a LINC00520 knockdown plasmid (MSC/shLINC00520) or the derived hucMSC-exosomes (EVs-MSC/shLINC00520) for 48 h. **B** Relative LINC00520 levels in hFOB1.19 cells exposed to hucMSCs transfected with a LINC00210 overexpression plasmid (MSC/LINC00520 OE) and the derived hucMSC-exosomes (EVs-MSC/LINC00520 OE) for 48 h. **C** Viability of hFOB1.19 cells treated with exosomes derived from hucMSCs transfected with a LINC00520 knockdown or LINC00520 overexpression plasmid. **D, E** Cell cycle analysis of hFOB1.19 cells treated with derived exosomes from hucMSCs transfected with a LINC00520 knockdown or LINC00520-overexpression plasmid. **F, G** Apoptosis of hFOB1.19 cells treated with derived exosomes from hucMSCs transfected with a LINC00520 knockdown or LINC00520-overexpression plasmid was analyzed using a FITC-Annexin V kit and flow cytometry

We next determined whether exosomes from hucMSCs transfected with shLINC00520 or LINC00520 OE regulate osteoblast proliferation. Co-culturing hFOB1.19 cells with hucMSCs transfected with shLINC00520 resulted in the inhibition of LINC00520 expression in hFOB1.19 cells (Fig. 5A). Similarly, after hFOB1.19 cells were treated with exosomes which were obtained from hucMSCs transfected with shLINC00520, the level of LINC00520 in hFOB1.19 cells was inhibited (Fig. 5A). Conversely, co-culturing hFOB1.19 cells with hucMSCs transfected with LINC00520 OE led to increased LINC00520 expression in hFOB1.19 cells (Fig. 5B). After hFOB1.19 cells were treated with exosomes which were obtained from hucMSCs transfected with LINC00520 OE, the level of LINC00520 in hFOB1.19 cells was increased (Fig. 5B). After hFOB1.19 cells were treated with exosomes from hucMSCs transfected with shLINC00520, the viability of hFOB1.19 cells was inhibited (Fig. 5C), and the cell cycle process of hFOB1.19 cells was arrested in the *G*_0_/*G*_1_ phase (Fig. 5D, E). When hFOB1.19 cells were treated with exosomes from hucMSCs transfected with LINC00520 OE, the viability of hFOB1.19 cells was promoted (Fig. 5C), and proportion of cells in the *G*_0_/*G*_1_ phase was decreased, indicating that cell cycle was accelerated (Fig. 5D, E). Compared with exosomes from hucMSCs transfected with shNC, exosomes from hucMSCs transfected with shLINC00520 promoted apoptosis of hFOB1.19 cells. However, there was no significant difference in the effect of exosomes from hucMSCs transfected with Vector and hucMSCs transfected with LINC00520 OE on the apoptosis of hFOB1.19 cells (Fig. 5F, G). Overall, these results suggest that manipulating LINC00520 expression in hucMSCs and subsequent treatment with their exosomes can influence the viability, cell cycle progression, and apoptosis of hFOB1.19 cells, highlighting the potential role of LINC00520 in osteoblast proliferation and function.

### LINC00520 in hucMSC-exosomes promotes the formation of calcified nodules in hFOB1.19 cells

**Fig. 6:**
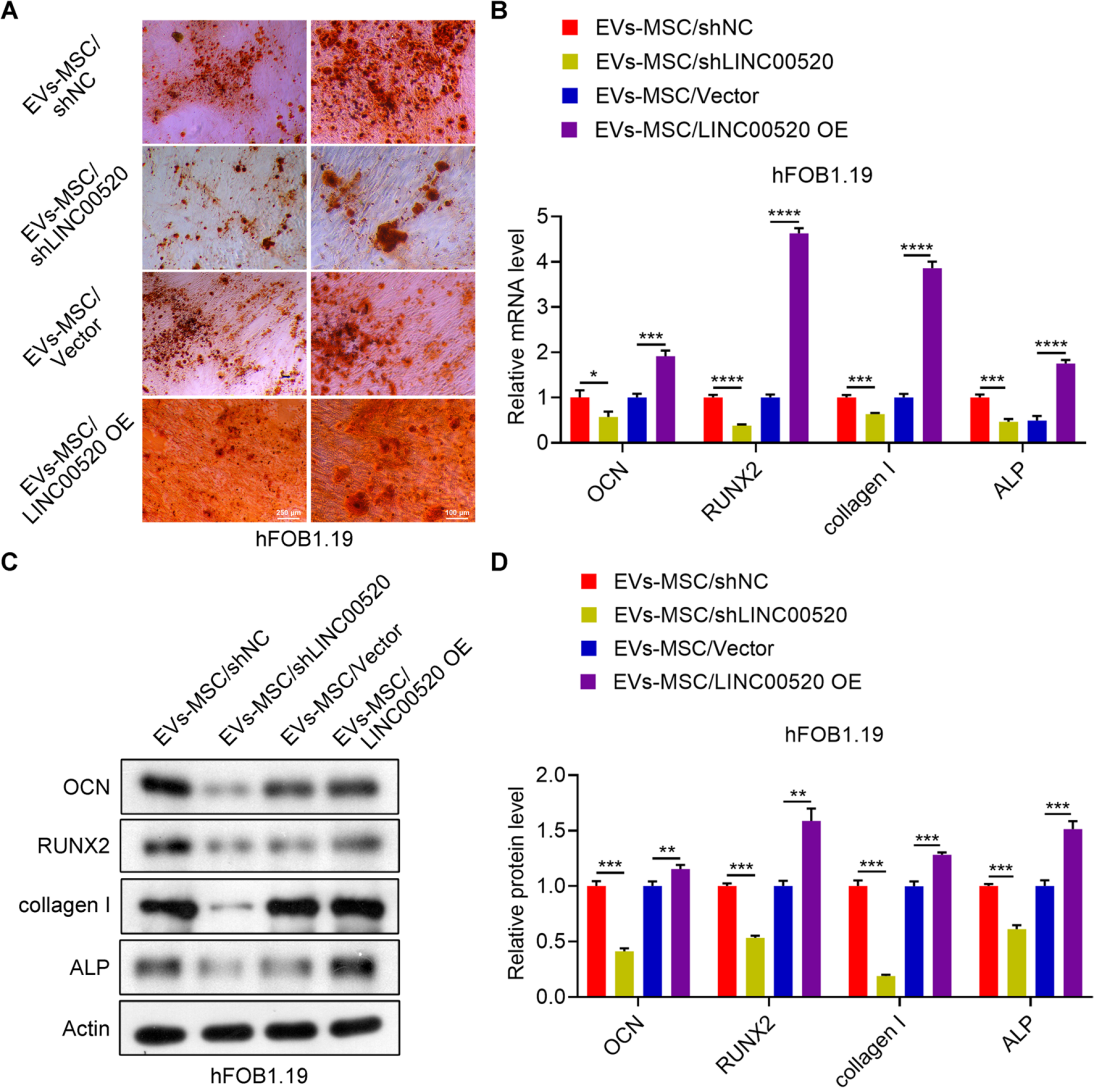
LINC00520 in hucMSC-exosomes promotes osteoblast differentiation. **A** Alizarin red staining of hFOB1.19 cells treated with exosomes derived from hucMSCs transfected with a LINC00520 knockdown or overexpression plasmid. **B** Relative mRNA expression levels of OCN, RUNX2, collagen I, and ALP in hFOB1.19 cells cultured with exosomes derived from hucMSCs transfected with a LINC00520 knockdown or overexpression plasmid. **C, D** Relative protein levels of OCN, RUNX2, collagen I, and ALP in hFOB1.19 cells cultured exosomes derived from hucMSCs transfected with a LINC00520 knockdown or overexpression plasmid

The above results show that LINC00520 has significant effects on osteoblast proliferation. Next, to determine whether LINC00520 affects the ability of hFOB1.19 cells to form mineralized bone, we treated hFOB1.19 cells with exosomes from hucMSCs transfected with shLINC00520 or LINC00520 OE plasmid and stained hFOB1.19 cells with alizarin red. Compared with the control cells, hFOB1.19 cells exposed to exosomes from hucMSCs transfected with shLINC00520 had fewer calcified nodules, whereas more alizarin red staining was observed in hFOB1.19 cells exposed to exosomes from hucMSCs transfected with LINC00520 OE (Fig. 6A). The mRNA and protein expression levels of OCN, RUNX2, collagen I and ALP were significantly decreased in hFOB1.19 cells cultured with exosomes from hucMSCs transfected with shLINC00520 and were significantly increased hFOB1.19 cells cultured with exosomes from hucMSCs transfected with LINC00520 OE (Fig. 6B, C, D).

## Discussion

Slowing or stopping the progression of osteoporosis remains a significant clinical challenge. Understanding the molecular mechanisms underlying osteoporosis is crucial for identifying new therapeutic candidates. Since exosomes have been shown to be involved in various pathological processes [29, 30], the possible role of exosomes in osteoporosis has drawn extensive attention in recent years. Many studies have shown that co-culture with hucMSC-exosomes can alleviate osteoporosis [12, 31, 32], and exosomes derived from MSCs efficiently induced bone regeneration [33–35]. These findings suggest the potential application of exosomes for osteoporosis treatment. However, a study has shown that exosomes aggravate osteoporosis [36]. Thus, the effects of exosomes on osteoporosis remain controversial. Here, we successfully isolated hucMSC-exosomes and found that low concentration of hucMSC-exosomes promoted hFOB1.19 cell proliferation while high concentration of exosomes inhibited hFOB1.19 cell proliferation. These results expand our understanding of the effects of hucMSC-exosomes in osteoporosis.

LINC00520, located on chromosome 14, is overexpressed in breast cancer, nasopharyngeal carcinoma and laryngeal squamous cell carcinoma and promotes the development of these cancers [37]. Studies have also shown that LINC00520 inhibit the growth and metastasis of cutaneous squamous cell carcinoma [38]. Our study was the first to link LINC00520 with osteoblast proliferation and differentiation and found that LINC00520 promotes the proliferation and differentiation of hFOB1.19 cells. In addition, knockdown of LINC00520 induced G1 phase arrest, which may be due to its participation in the PI3k/AKT pathway [38]. Similarly, exosomes from hucMSCs with LINC00520 knockdown cause cell cycle arrest in hFOB1.19 cells in G1 phase.

In recent years, many studies have shown that LINC00520 is involved in the development and progression of disease by targeting multiple miRNAs, such as miR-4516, miR-1252-5p and miR-577 [39–41]. LINC00520 upregulates SOX5, FOXR2, or EIF5A2 through its interaction with miR-4516, miR-1252-5p, or miR-125b-5p, respectively [37, 39, 40]. This regulatory mechanism promotes cell proliferation and invasion in human hepatocellular carcinoma, accelerates the progression of lung adenocarcinoma, and enhances the proliferation and metastasis of malignant melanoma. Furthermore, LINC00520 increases the expression of POSTN, CCNE2, or HSP27 by targeting miR-577, thereby accelerating the progression of breast cancer, non-small cell lung cancer, or colorectal cancer [41–43]. In addition, LINC00520 promotes the development of acute kidney injury by targeting miR-27b-3p and regulating OSMR expression through the PI3K/AKT signaling pathway [44]. In this study, we found that exosomes from hucMSCs overexpressing LINC00520 promoted hFOB1.19 cell survival and the ability to form mineralized bone. Whether LINC00520 regulates mineralized bone-related genes in osteoblasts by targeting miRNAs remains to be further investigated.

This study has several limitations. First, because of the complexity of the exosome secretion and transmission mechanisms, further studies are necessary to confirm their function. Second, we only used hFOB1.19 cells to explore the effect of hucMSC-exosomes on osteoblast proliferation and osteogenic capacity, and an animal model was needed to further verify our findings [45].

## Conclusions

In summary, hucMSC-exosomes regulate the proliferation and differentiation of osteoblasts via LINC00520. In hFOB1.19 cells, exosomes from hucMSCs down-regulated LINC00520 decreased cell viability and ability to form mineralized bone, blocked cell cycles, and increased apoptosis. Exosomes from hucMSCs overexpressing LINC00520 enhanced cell viability and the cell's ability to form mineralized bone and accelerated the cell cycle. These findings suggest that exosomes from hucMSCs overexpressing LINC00520 may be a novel therapeutic approach for osteoporosis.

## Supplementary Information

**Additional file 1:** The original images of all immunobloting in the study.

## Data Availability

LncRNA data from multiple myeloma-derived MSC was obtained from the gene expression omnibus (GSE118985, https://www.ncbi.nlm.nih.gov/geo/query/acc.cgi?acc=GSE118985). lncRNAs from exosome exoRBase (http://www.exorbase.org/) was used to predict lncRNAs regulating osteoblast differentiation in exosomes. Other datasets used or analyzed during this study are available from the corresponding author on reasonable request.
