# Supplementary material for: Exosomes promote hFOB1.19 proliferation and differentiation via LINC00520
Source: J Orthop Surg Res. 2023 Jul 29;18:546. doi: 10.1186/s13018-023-04021-y (PMC10387216; doi:10.1186/s13018-023-04021-y)
Supplement: Supplementary file 1 — Additional file 1: The original images of all immunobloting in the study. [file 13018_2023_4021_MOESM1_ESM.pdf]

Figure 1F: Protein levels of CD9, CD81, and CD63 in exosomes

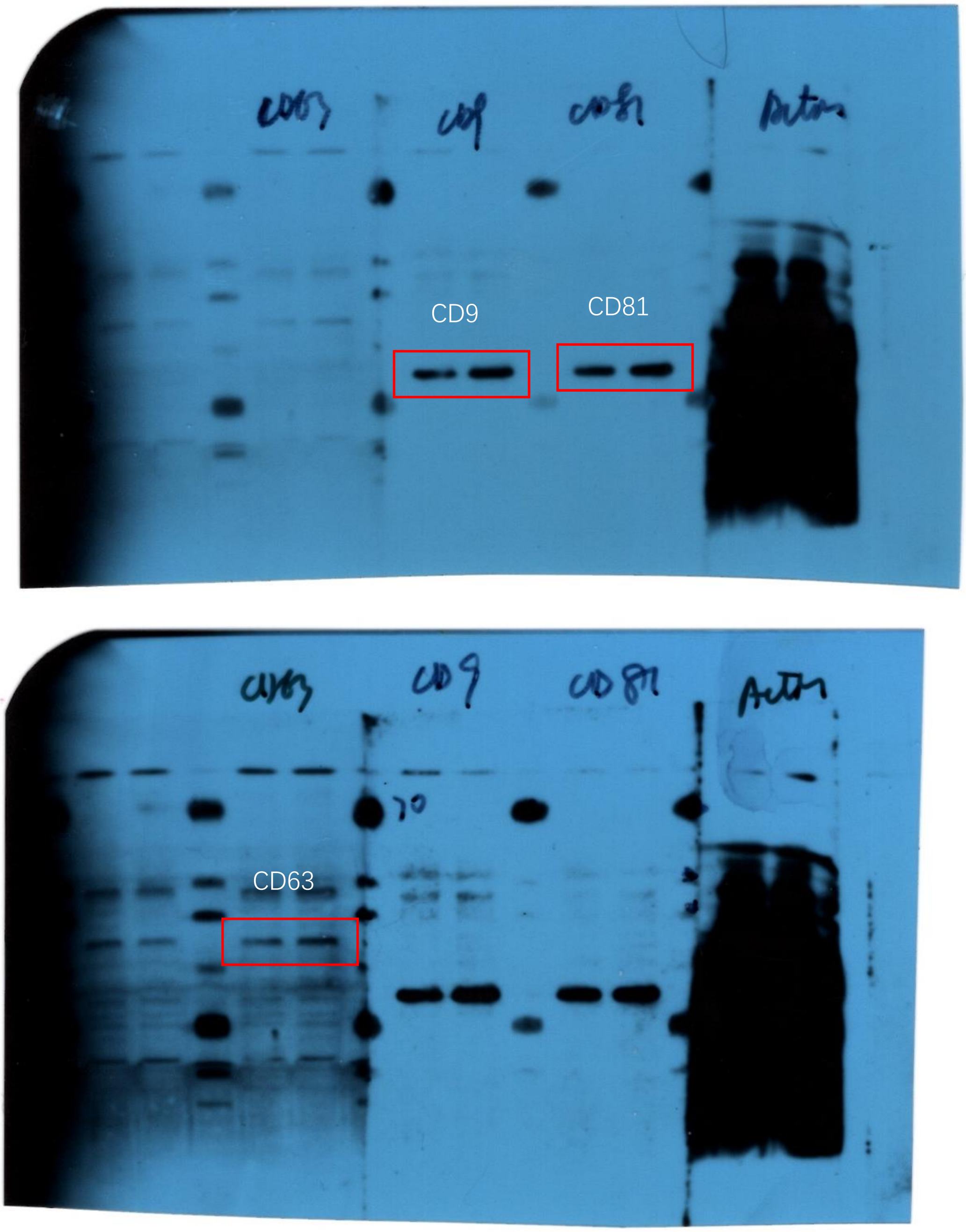

Figure3C: Protein levels of OCN, RUNX2, collagen I and ALP in hFOB1.19 cells cocultured with hucMSC-exosomes or hucMSC.

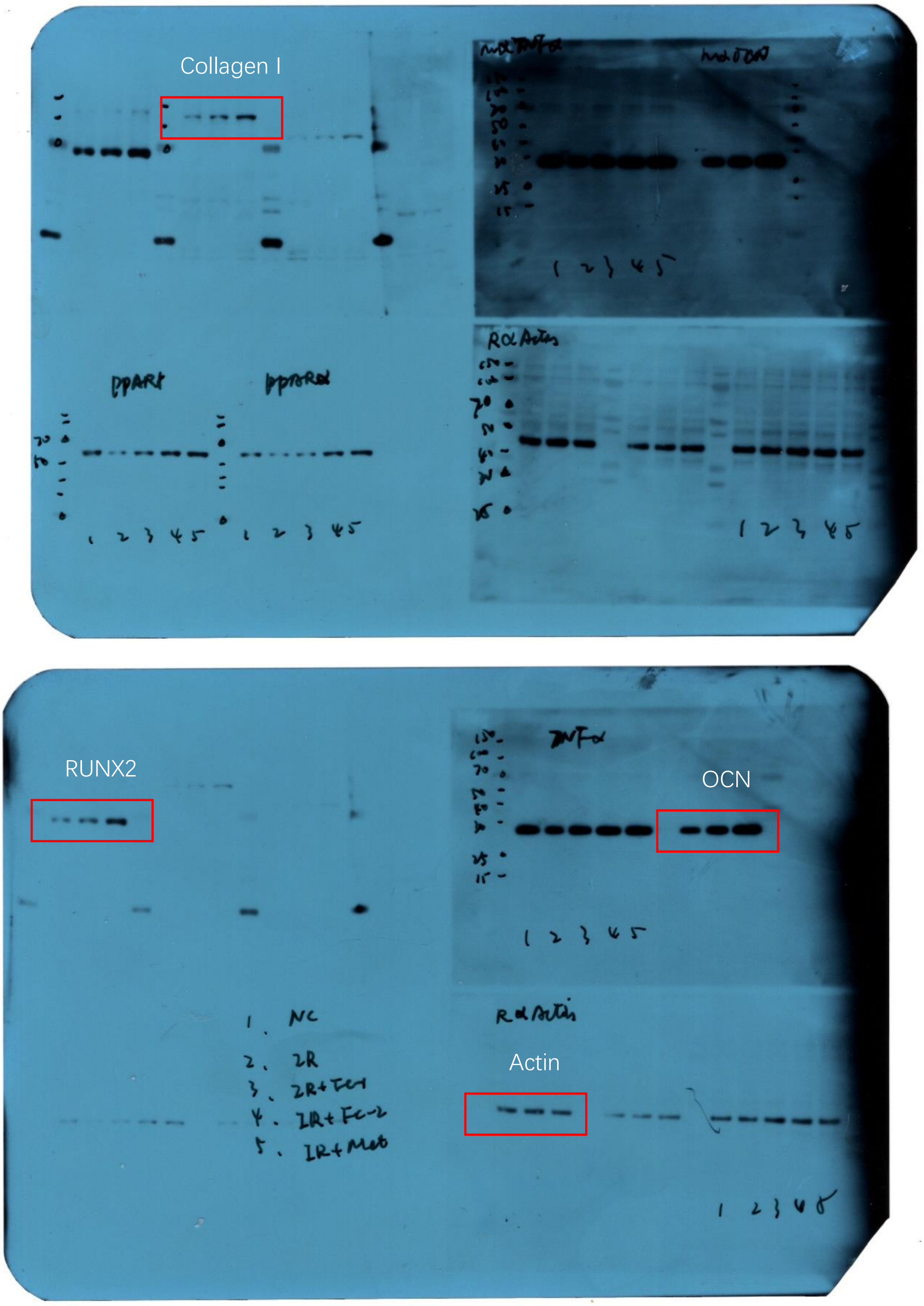

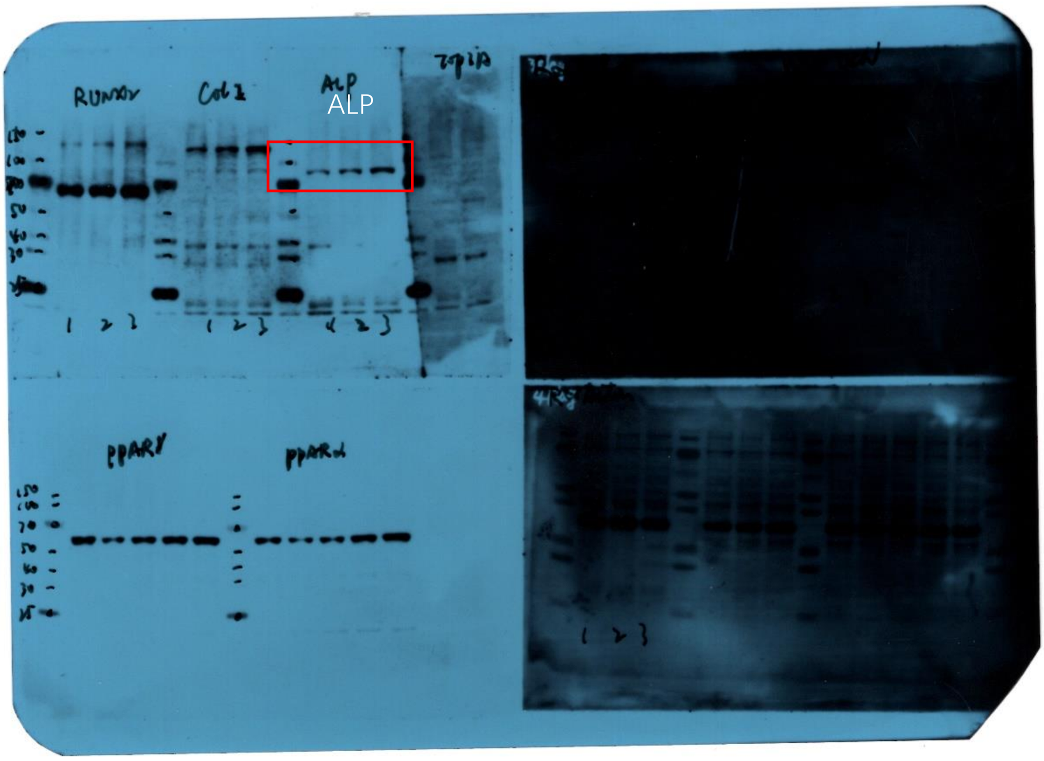
ALP

Figure 6C: Protein levels of OCN, RUNX2, collagen I and ALP in hFOB1.19 cells cocultured with exosomes from hucMSCs transfected plasmids (shLINC00520 or LINC00520 OE).

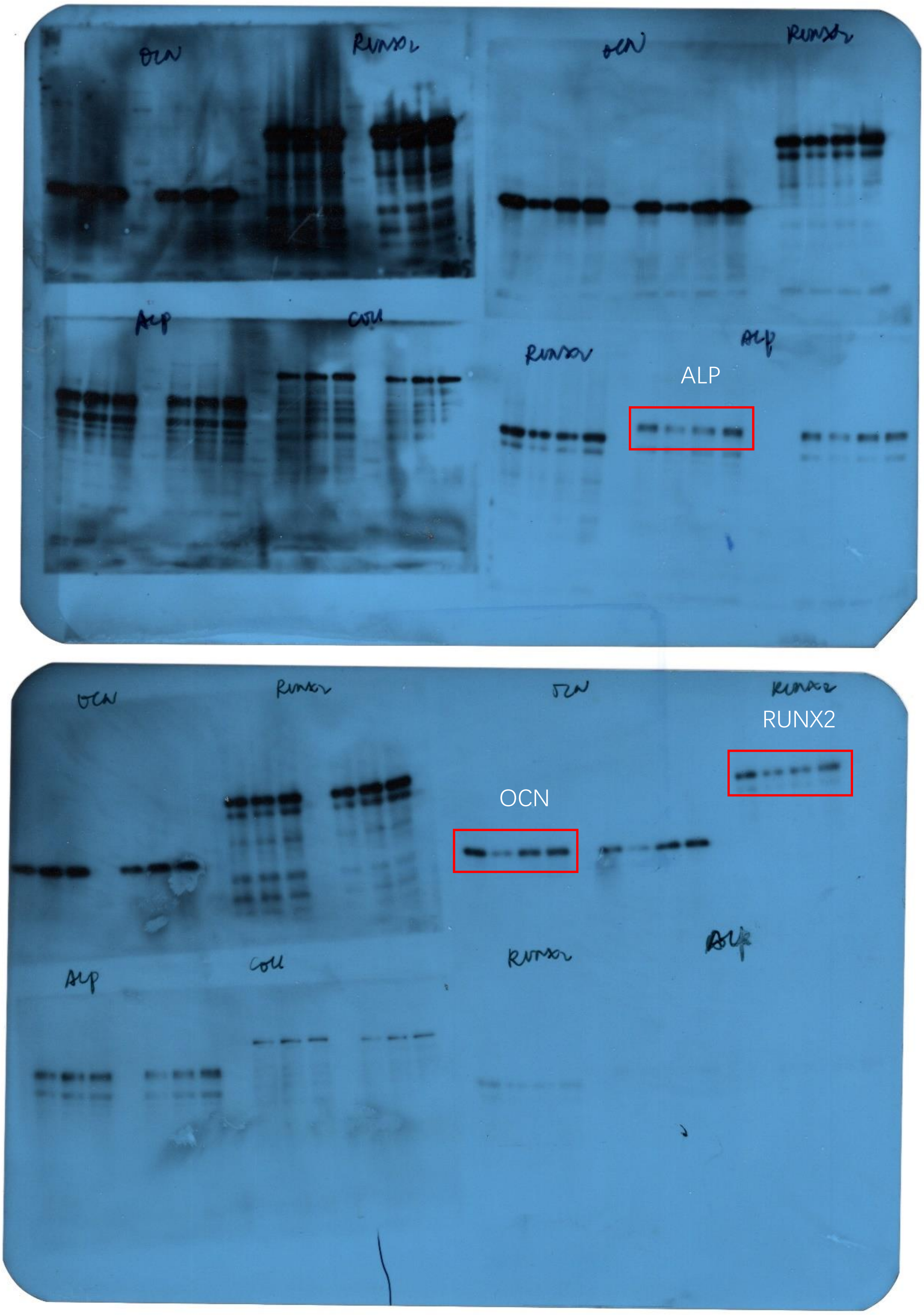

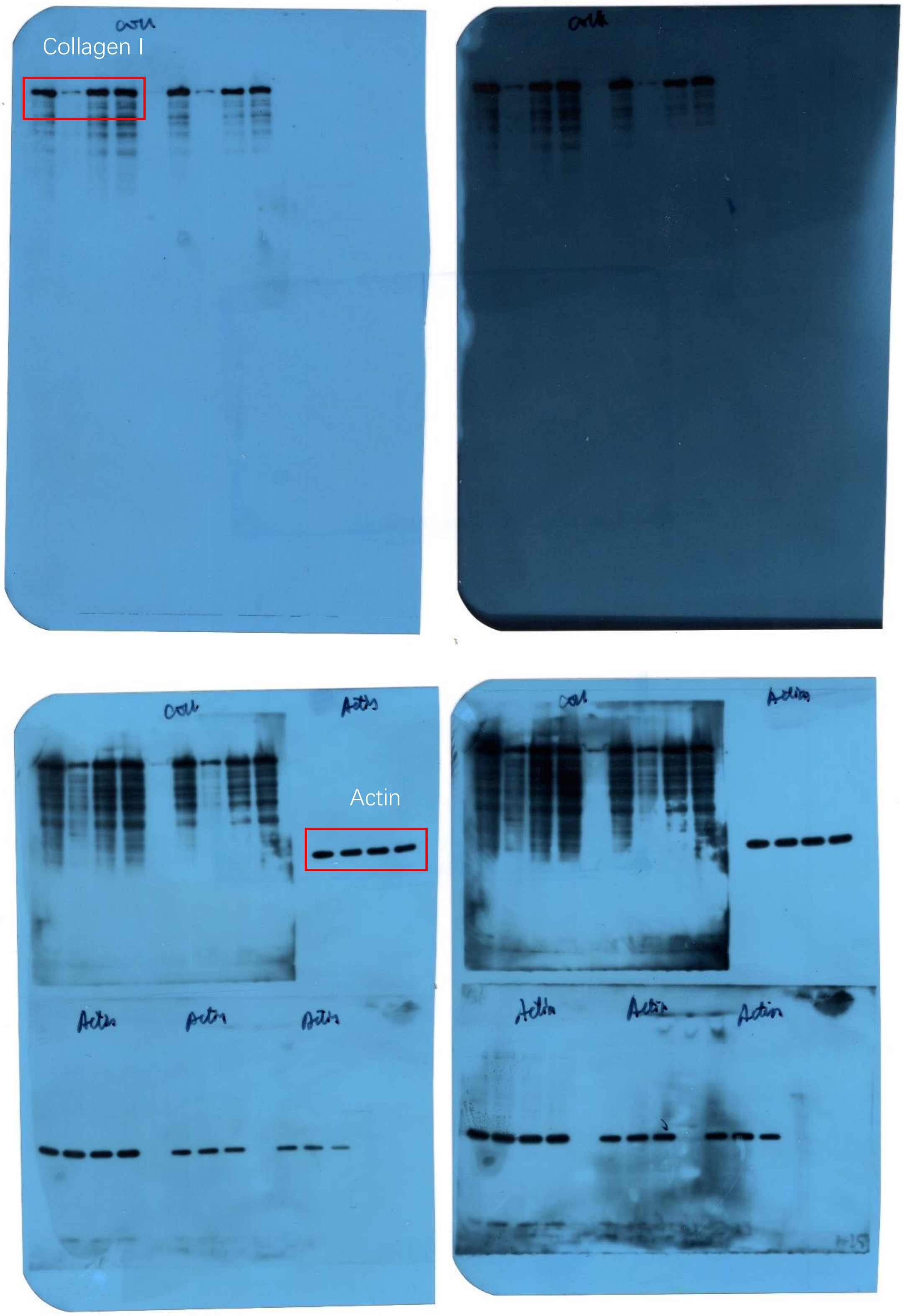
Collagen I
Actin
